# Prevalence and Disparities in Telehealth Use Among US Adults Following the COVID-19 Pandemic: National Cross-Sectional Survey

**DOI:** 10.2196/52124

**Published:** 2024-05-10

**Authors:** Erin M Spaulding, Michael Fang, Yvonne Commodore-Mensah, Cheryl R Himmelfarb, Seth S Martin, Josef Coresh

**Affiliations:** 1 Johns Hopkins University School of Nursing Baltimore, MD United States; 2 Digital Health Innovation Laboratory Ciccarone Center for the Prevention of Cardiovascular Disease, Division of Cardiology Department of Medicine, Johns Hopkins University School of Medicine Baltimore, MD United States; 3 Welch Center for Prevention, Epidemiology, and Clinical Research Johns Hopkins Bloomberg School of Public Health Baltimore, MD United States; 4 Department of Epidemiology Johns Hopkins Bloomberg School of Public Health Baltimore, MD United States; 5 Center for Health Equity Johns Hopkins University Baltimore, MD United States; 6 Johns Hopkins University Whiting School of Engineering Baltimore, MD United States; 7 Optimal Aging Institute New York University Grossman School of Medicine New York, NY United States; 8 Division of Epidemiology Department of Population Health New York University Grossman School of Medicine New York, NY United States

**Keywords:** telehealth, telemedicine, delivery of health care, health care disparities, COVID-19

## Abstract

**Background:**

Telemedicine expanded during the COVID-19 pandemic, though use differed by age, sex, race or ethnicity, educational attainment, income, and location. It is unclear if high telehealth use or inequities persisted late into the pandemic.

**Objective:**

This study aims to evaluate the prevalence of, inequities in, and primary reasons for telehealth visits a year after telemedicine expansion.

**Methods:**

We used cross-sectional data from the 2022 Health Information National Trends Survey (HINTS 6), the first cycle with data on telemedicine. In total, 4830 English- and Spanish-speaking US adults (aged ≥18 years) were included in this study. The primary outcomes were telehealth visit attendance in the 12 months before March 7, 2022, to November 8, 2022, and the primary reason for the most recent telehealth visit. We evaluated sociodemographic and clinical predictors of telehealth visit attendance and the primary reason for the most recent telehealth visit through Poisson regression. Analyses were weighted according to HINTS 6 standards.

**Results:**

We included 4830 participants (mean age 48.3, SD 17.5 years; 50.28% women; 65.21% White). Among US adults, 38.78% reported having a telehealth visit in the previous year. Telehealth visit attendance rates were similar across age, race or ethnicity, income, and urban versus rural location. However, individuals with a telehealth visit were less likely to live in the Midwest (adjusted prevalence ratio [aPR] 0.65, 95% CI 0.54-0.77), and more likely to be women (aPR 1.21, 95% CI 1.06-1.38), college graduates or postgraduates (aPR 1.24, 95% CI 1.05-1.46), covered by health insurance (aPR 1.56, 95% CI 1.08-2.26), and married or cohabitating (aPR 1.17, 95% CI 1.03-1.32), adjusting for sociodemographic characteristics, frequency of health care visits, and comorbidities. Among participants with a telehealth visit in the past year, the primary reasons for their most recent visit were minor or acute illness (32.15%), chronic disease management (21%), mental health or substance abuse (16.94%), and an annual exam (16.22%). Older adults were more likely to report that the primary reason for their most recent telehealth visit was for chronic disease management (aPR 2.08, 95% CI 1.33-3.23), but less likely to report that it was for a mental health or substance abuse issue (aPR 0.19, 95% CI 0.10-0.35), adjusting for sociodemographic characteristics and frequency of health care visits.

**Conclusions:**

Among US adults, telehealth visit attendance was high more than a year after telemedicine expansion and did not differ by age, race or ethnicity, income, or urban versus rural location. Telehealth could continue to be leveraged following COVID-19 to improve access to care and health equity.

## Introduction

Access to health care, or the timely use of personal health services, is critical to achieving the best possible health outcomes [1,2]. However, many individuals face barriers to obtaining care, such as a lack of health insurance, poor access to transportation, and appointment times only being offered during typical work hours [2,3]. Telehealth has the potential to improve access to care in the United States. The Centers for Medicare and Medicaid Services define telehealth as real-time communication between a patient and a physician or practitioner through telecommunications equipment, including synchronous (both audio-video and audio-only) visits [4]. Previously, telemedicine coverage was largely limited to individuals living in rural areas and had to be conducted by video in a health care facility [5]. In response to COVID-19, on March 17, 2020, the Centers for Medicare and Medicaid Services temporarily expanded telemedicine, enabling beneficiaries to receive services outside a health care facility [6].

Studies using claims and electronic health record data demonstrated that telehealth use increased significantly from the prepandemic through the pandemic period [7-11]. While telemedicine expansion can improve access to health care, it also has the potential to exacerbate existing disparities among individuals with limited digital access and literacy. As such, digital access and literacy are increasingly being recognized as overarching social determinants of health [12]. Data from the 2018 American Community Survey (ACS) demonstrated that 26% of Medicare beneficiaries lacked digital access at home (ie, were without a computer or internet or a smartphone or data plan) and were likely unprepared for the dramatic shift in care toward telemedicine [13]. This was especially true among older adults, non-Hispanic Black and Hispanic adults, widowed individuals, adults with a high school education or less, adults with an income 200% below the federal poverty level, adults enrolled in Medicaid, and those with a disability [13].

Cross-sectional data between March 19, 2020, and March 24, 2020, from the Pew Research Center’s American Trends Panel of US adults, immediately following the temporary expansion of telemedicine, demonstrated that approximately 17% of individuals reported using telehealth, with use being higher among racial and ethnic minorities [14]. The 2021 National Health Interview Survey (NHIS) data demonstrated that 37% of adults used telemedicine in the 12 months before January 2021-December 2021, during the early stages of COVID-19 [15]. Significant disparities in telehealth use were found, with older adults, women, non-Hispanic White and non-Hispanic American Indian or Alaska Native adults, adults with higher income and education, and adults living in urban areas and in the Northeast and West of the United States being more likely to have used telehealth in the past year [15]. Studies using claims and electronic health record data also found disparities in telehealth use during the early stages of the pandemic [8-11,16-18]. Understanding the prevalence and predictors of telehealth visit attendance nationally, beyond the first year of the COVID-19 pandemic, could aid in decision-making around the continued use of telehealth to improve access to care.

We used 2022 Health Information National Trends Survey (HINTS 6) data to evaluate the prevalence of and sociodemographic and clinical predictors of telehealth visit attendance in the 12 months before March 2022-November 2022 and the primary reasons for the most recent telehealth visits.

## Methods

### Data Source and Study Population

This study used publicly available cross-sectional data from the 2022 HINTS 6 data set administered by the National Cancer Institute. HINTS 6 is a nationally representative survey of English- and Spanish-speaking civilian, noninstitutionalized adults living in the United States. Participants could respond to the survey on paper or the web [19]. HINTS 6, conducted between March 7, 2022, and November 8, 2022, is the first survey cycle to ask questions about telehealth visits [19]. The sampling strategy consisted of a two-stage design: (1) a stratified sample of addresses was selected from a database of residential addresses in the United States, and (2) one adult was selected within each sampled household [19].

The sampling frame of addresses was grouped into four sampling strata: (1) high minority urban, (2) low minority urban, (3) high minority rural, and (4) low minority rural [19]. The high minority stratum was defined to include addresses in census tracts from the 2015-2019 ACS with at least a 34% population proportion of African Americans or Hispanics [19,20]. The rural or urban strata were based on the latest 2013 US Department of Agriculture, Economic Research Service, rural-urban continuum codes [19,21]. The overall weighted response rate, calculated using the Response Rate 4 formula of the American Association of Public Opinion Research [22], was 28% and differed by strata (high minority urban: 21%, low minority urban: 31%, high minority rural: 22%, and low minority rural: 29%) [19]. The high minority strata were oversampled, and within the low minority strata, the rural stratum was sampled at a higher rate than the urban stratum [19]. Survey weights were demographically calibrated using 2021 ACS estimates (age, sex, educational attainment, marital status, race, ethnicity, and census region) and HINTS 6–reported insurance and cancer status [19]. We included participants in this analysis with data on telehealth use, sociodemographics, frequency of health care visits, and comorbidities.

### Outcome Measures

All participants were asked “In the past 12 months, did you receive care from a doctor or health professional using telehealth?” to assess telehealth visit attendance from March 2022 to November 2022 [23]. A telehealth visit was defined as a “telephone or video appointment with a doctor or health professional” [23].

Participants with a telehealth visit in the past 12 months were asked “What was the primary reason for your most recent telehealth visit?” [23]. Response options include annual visit; minor illness or acute care (eg, fever or sinus infection); managing chronic condition or disease (eg, high blood pressure, diabetes, heart disease, obesity, or cancer); medical emergency; mental health, behavioral, or substance abuse issue (eg, depression, anxiety, or drug or alcohol abuse); or other [23].

### Covariates

Covariates included age, sex, race or ethnicity, education, annual household income, health insurance, marital status, location, census region, number of times in the past 12 months the participant received care from a health professional (excluding emergency room visits), and comorbidities (diabetes; hypertension; heart condition [heart attack, angina, or heart failure]; chronic lung disease, asthma, emphysema, or chronic bronchitis; and depression or anxiety disorder). Education level was categorized as high school graduate or less, some college or vocational or technical school, or college graduate or postgraduate. Annual household income was categorized as ≤US $34,999, US $35,000-US $74,999, or ≥US $75,000. Health insurance was recorded as being covered or not covered by any kind of health insurance plan. Marital status was categorized as married or cohabitating or divorced, widowed, separated, or single as a proxy for social support. Location was categorized as urban (metropolitan) or rural (nonmetropolitan). The census region was categorized as Northeast, Midwest, South, or West. The number of times the participant received care from a health professional in the past 12 months was dichotomized into less than 5 times and 5 times or more.

### Statistical Analysis

We estimated the proportion of US adults with a telehealth visit in the past 12 months and the primary reason for their most recent telehealth visit. We used recommended methods for HINTS 6, including survey weights and the delete one jackknife replication method, to account for the complex survey design and generate nationally representative estimates [19,24]. This jackknife variance estimation technique deleted one primary sampling unit at a time from the full sample to create a set of 50 replicate weights [19,24]. We examined prevalence ratios of sociodemographic and clinical predictors of telehealth visit attendance in the past 12 months and their primary reason for their most recent telehealth visit using generalized linear models with a Poisson distribution and logarithmic link. Prevalence ratios are the preferred statistical estimate for cross-sectional studies when the outcome or outcomes are not rare [25]. The reported percentages are weighted and the sample sizes are unweighted. Analyses were conducted using Stata (version 18.0; StataCorp).

### Ethical Considerations

HINTS 6 (2022) was given a nonhuman subjects research designation from the National Institutes of Health Office of Human Subjects Research Protections on August 16, 2021. It was reviewed and approved by the Westat Institutional Review Board on May 10, 2021, with a subsequent amendment reviewed and approved on November 24, 2021 (6632.03.51). Thus, informed consent was not required for the primary data collection. This analysis using HINTS 6 data also met criteria for nonhuman subject research by the Johns Hopkins University School of Medicine institutional review board. Thus, ethical review and approval were not needed for this study because it involves secondary data analysis of existing, deidentified, publicly available data.

## Results

This study included 4830 participants, of which the mean age was 48.3 (SD 17.5) years, 50.28% (2886/4830) were women, 65.21% (2895/4830) were non-Hispanic White adults, 39.51% (1367/4830) had some college or vocational or technical schooling, 45.88% (1991/4830) had an annual household income of at least US $75,000, 89.59% (4437/4830) had health insurance, 56.88% (2573/4830) were married or cohabitating, 88.23% (4225/4830) lived in an urban location, 38.4% (2176/4830) lived in the South, and 78.25% (3589/4830) had less than 5 visits with a health professional in the previous 12 months. Survey-weighted characteristics are provided in Table 1.

**Table 1:** Predictors of telehealth visits in 12 months before Health Information National Trends Survey (HINTS 6; March 2022-November 2022) among weighted US adults (N=4830). Weights are calibrated using data from the 2021 American Community Survey (age, sex, educational attainment, marital status, race, ethnicity, and census region) conducted by the US Census Bureau.

Sociodemographic characteristics			US adults weighted % (95% CI)^a^		Telehealth visit in past 12 months, weighted % (95% CI)^b^		Unadjusted PR^c^ (95% CI)		Model 1 adjusted PR (95% CI)^d^		Model 2 adjusted PR (95% CI)^e^
Overall			N/A^f^		38.78 (36.68-40.91), n=1986		N/A		N/A		N/A
**Age (years)**											
	18-44	42.70 (40.91-44.51)		37.9 (34.03-41.93)		1 (reference)		1 (reference)		1 (reference)	
	45-64	37.47 (35.69-39.29)		40.75 (37.21-44.38)		1.08 (0.94-1.23)		1.04 (0.91-1.19)		1.02 (0.89-1.18)	
	≥65	19.83 (19.06-20.62)		36.93 (33.71-40.27)		0.97 (0.83-1.14)		0.90 (0.76-1.06)		0.87 (0.73-1.03)	
**Sex**											
	Male	49.72 (48.44-51)		33.47 (30.42-36.67)		1 (Reference)		1 (Reference)		1 (Reference)	
	Female	50.28 (49-51.56)		44.02 (40.83-47.26)		1.32 (1.16-1.49)^g^		1.29 (1.14-1.47)^g^		1.21 (1.06-1.38)^h^	
**Race or ethnicity**											
	Non-Hispanic White	65.21 (64.36-66.05)		39.68 (36.79-42.65)		1 (Reference)		1 (Reference)		1 (Reference)	
	Non-Hispanic Black	11.48 (10.9-12.08)		33.66 (28.18-39.62)		0.85 (0.70-1.03)		0.89 (0.73-1.07)		0.98 (0.82-1.17)	
	Hispanic	17.24 (16.47-18.04)		38.84 (33.55-44.41)		0.98 (0.83-1.16)		1 (0.83-1.22)		1.11 (0.92-1.34)	
	Non-Hispanic Asian	6.08 (5.53-6.68)		38.52 (26.41-52.26)		0.97 (0.68-1.38)		0.89 (0.65-1.23)		1.05 (0.77-1.42)	
**Education**											
	High school graduate or less	26.87 (25.13-28.68)		33.79 (29.68-38.17)		1 (reference)		1 (reference)		1 (reference)	
	Some college or vocational or technical school	39.51 (37.93-41.12)		37.22 (33.58-41.01)		1.10 (0.94-1.30)		1.09 (0.92-1.29)		1.09 (0.91-1.30)	
	College graduate or postgraduate	33.62 (32.79-34.46)		44.59 (41.45-47.77)		1.32 (1.15-1.52)^g^		1.22 (1.03-1.44)^h^		1.24 (1.05-1.46)^h^	
**Annual household income (US $)**											
	≤34,999	23.75 (21.69-25.94)		36.13 (31.24-41.32)		1 (reference)		1 (reference)		1 (reference)	
	35,000-74,999	30.37 (28.26-32.56)		34.79 (30.84-38.96)		0.96 (0.80-1.16)		0.92 (0.76-1.13)		1.01 (0.84-1.21)	
	≥75,000	45.88 (43.51-48.27)		42.78 (38.85-46.8)		1.18 (0.99-1.42)		1 (0.81-1.23)		1.13 (0.92-1.38)	
**Health insurance**											
	No	10.41 (9.57-11.32)		22.15 (15.21-31.1)		1 (reference)		1 (reference)		1 (reference)	
	Yes	89.59 (88.68-90.43)		40.71 (38.31-43.15)		1.84 (1.25-2.70)^h^		1.73 (1.16-2.59)^h^		1.56 (1.08-2.26)^h^	
**Marital status**											
	Divorced, widowed, separated, or single	43.12 (41.82-44.44)		35.39 (31.67-39.29)		1 (reference)		1 (reference)		1 (reference)	
	Married or cohabitating	56.88 (55.56-58.18)		41.34 (38.8-43.94)		1.17 (1.03-1.33)^h^		1.12 (0.98-1.27)		1.17 (1.03-1.32)^h^	
**Location**											
	Urban	88.23 (87.34-89.07)		39.21 (37.05-41.42)		1 (reference)		1 (reference)		1 (reference)	
	Rural	11.77 (10.93-12.66)		35.49 (30.09-41.29)		0.91 (0.77-1.06)		0.97 (0.81-1.15)		0.93 (0.80-1.10)	
**Census region**											
	Northeast	17.55 (16.69-18.44)		45.16 (39.7-50.73)		1 (reference)		1 (reference)		1 (reference)	
	Midwest	21.8 (20.87-22.75)		27.9 (24.16-31.98)		0.62 (0.51-0.74)^g^		0.63 (0.52-0.76)^g^		0.65 (0.54-0.77)^g^	
	South	38.4 (37.37-39.43)		37.63 (34.11-41.29)		0.83 (0.71-0.98)^h^		0.88 (0.76-1.03)		0.87 (0.74-1.01)	
	West	22.26 (21.33-23.21)		46.36 (41.76-51.02)		1.03 (0.89-1.19)		1.06 (0.91-1.23)		1.05 (0.89-1.23)	
**Frequency of visits with health professional**											
	<5	78.25 (76.18-80.2)		33.56 (30.99-36.23)		1 (reference)		N/A		1 (reference)	
	≥5	21.75 (19.8-23.82)		57.55 (53.59-61.42)		1.72 (1.54-1.91)^g^		N/A		1.47 (1.32-1.63)^g^	
**Diabetes**											
	No	83.63 (82.01-85.13)		37.36 (35.21-39.56)		1 (reference)		N/A		1 (reference)	
	Yes	16.37 (14.87-17.99)		45.99 (40.18-51.91)		1.23 (1.07-1.41)^h^		N/A		1.09 (0.96-1.25)	
**Hypertension**											
	No	63.51 (61.34-65.63)		36.57 (33.53-39.73)		1 (reference)		N/A		1 (reference)	
	Yes	36.49 (34.37-38.66)		42.61 (38.77-46.55)		1.17 (1.01-1.34)^h^		N/A		1.12 (0.97-1.31)	
**Heart condition**											
	No	92.94 (91.84-93.9)		37.87 (35.67-40.11)		1 (reference)		N/A		1 (reference)	
	Yes	7.06 (6.1-8.16)		50.73 (42.97-58.45)		1.34 (1.14-1.58)^h^		N/A		1.20 (1.02-1.42)^h^	
**Lung disease**											
	No	88.51 (87.17-89.74)		37.41 (34.98-39.92)		1 (reference)		N/A		1 (reference)	
	Yes	11.49 (10.26-12.83)		49.27 (44.13-54.43)		1.32 (1.15-1.51)^g^		N/A		1.06 (0.91-1.23)	
**Depression or anxiety**											
	No	71.92 (69.55-74.18)		31.75 (29.48-34.1)		1 (reference)		N/A		1 (reference)	
	Yes	28.08 (25.82-30.45)		56.77 (52.16-61.27)		1.79 (1.60-2)^g^		N/A		1.65 (1.47-1.86)^g^	

^a^US adults’ characteristics are weighted column percentages.

^b^Telehealth visit in past 12 months by sociodemographic characteristics are weighted row percentages.

^c^PR: prevalence ratio.

^d^Model 1: adjusting for age, sex, race or ethnicity, education, income, health insurance, marital status, location, and census region.

^e^Model 2: adjusting for age, sex, race or ethnicity, education, income, health insurance, marital status, location, census region, frequency of visits with a health professional, and comorbidities.

^f^Not applicable.

^g^*P*<.001.

^h^*P*<.05.

The weighted prevalence of telehealth visit attendance in the past 12 months was 38.78% (1986/4830, 95% CI 36.68%-40.91%). Telehealth visit attendance in the past 12 months was similar by age (aged between 18 and 44 years: 634/1456, 37.9%; aged between 45 and 64 years: 729/1738, 40.75%; ≥65 years: 623/1636, 36.93%), race or ethnicity (non-Hispanic White: 1198/2895, 39.68%; non-Hispanic Black: 298/787, 33.7%; Hispanic: 393/885, 38.8%; non-Hispanic Asian: 97/263, 38.5%), annual household income (≤US $34,999: 517/1367, 36.13%; US $35,000-US $74,999: 570/1472, 34.79%; ≥US $75,000: 899/1991, 42.78%), and location (urban: 1782/4225, 39.21%; rural: 204/605, 35.5%). However, individuals who reported having a telehealth visit in the past 12 months were less likely to live in the Midwest (266/834, 22.9% vs 312/715, 45.2% Northeast; adjusted prevalence ratio [aPR] 0.65, 95% CI 0.54-0.77), and more likely to be women (1276/2886, 44.02% vs 710/1944, 33.47% men; aPR 1.21, 95% CI 1.06-1.38), college graduates or postgraduates (1081/2362, 44.59% vs 362/1101, 33.79% high school graduate or less; aPR 1.24, 95% CI 1.05-1.46), covered by health insurance (1872/4437, 41% vs 114/393, 22.2% not covered; aPR 1.56, 95% CI 1.08-2.26), married or cohabitating (1096/2573, 41.34% vs 890/2257, 35.4% divorced, widowed, separated, or single; aPR 1.17, 95% CI 1.03-1.32), have 5 or more visits in the previous 12 months with a health professional (700/1241, 57.55% vs 1286/3589, 33.56% with less than 5 visits; aPR 1.47, 95% CI 1.32-1.63), have a heart condition (224/451, 50.7% vs 1762/4379, 37.87%; aPR 1.20, 95% CI 1.02-1.42), and have depression or an anxiety disorder (753/1317, 56.77% vs 1233/3513, 31.75%; aPR 1.65, 95% CI 1.47-1.86), after adjusting for sociodemographic characteristics, frequency of visits with a health professional, and comorbidities (Table 1). While individuals with diabetes, hypertension, and lung disease were more likely to have reported a telehealth visit in the past 12 months in unadjusted models, this did not hold true after adjusting for other sociodemographic characteristics, frequency of visits with a health professional, and comorbidities. Similarly, adults living in the South were less likely to have reported a telehealth visit in the past 12 months in the unadjusted model but not in the adjusted models.

**Figure 1:**
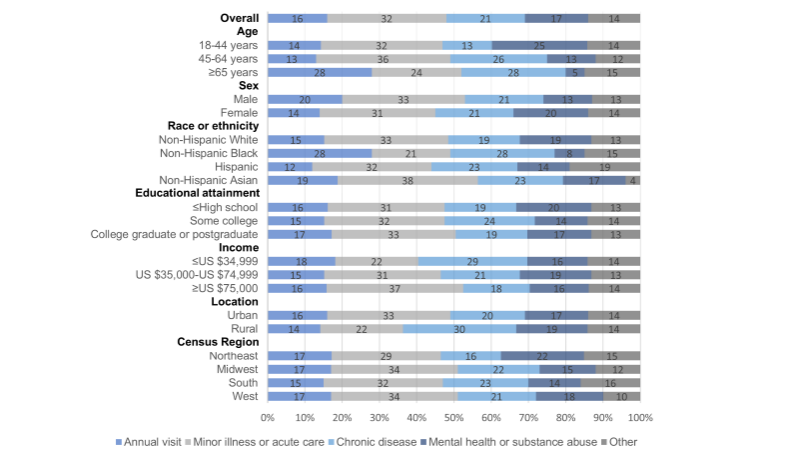
Prevalence of the primary reasons for telehealth visits in the 12 months before the Health Information National Trends Survey (HINTS 6; March 2022-November 2022) among weighted US adults (n=1878). Estimates are based on HINTS 6 data fielded from March through November 2022. Question text: “What was the primary reason for your most recent telehealth visit?” Response options (select 1): annual visit, minor illness or acute care (eg, fever or sinus infection), managing my chronic health condition or disease (eg, high blood pressure, diabetes, heart disease, obesity, or cancer), mental health, behavioral or substance abuse issues (eg, depression, anxiety, or drug or alcohol abuse), medical emergency, or other. This question was asked of participants who reported having a telehealth visit in the past 12 months.

The primary reasons for participants’ most recent telehealth visit in the past 12 months (Figure 1; N=1878) were for a minor illness or acute care (510/1878, 32.15%, 95% CI 28.1%-36.49%), managing a chronic disease (462/1878, 21%, 95% CI 18.27%-24.03%), mental health, behavioral or substance abuse issues (288/1878, 16.94%, 95% CI 14.15%-20.16%), annual visits (352/1878, 16.22%, 95% CI 13.62%-19.2%), and other reasons (266/1878, 13.68%, 95% CI 11.26%-16.53%). Individuals who reported that the primary reason for their most recent telehealth visit in the past year was for an annual visit were more likely to be aged 65 years and older (158/560, 28.3% vs 93/622, 14.5% aged between 18 and 44 years; aPR 2.16, 95% CI 1.52-3.08) and non-Hispanic Black adults (83/271, 28.2% vs 189/1145, 15.39% White; aPR 1.96, 95% CI 1.28-3), and less likely to be women (195/1206, 13.6% vs 157/672, 19.7% men; aPR 0.68, 95% CI 0.50-0.93) and have had 5 or more visits with a health professional in the previous 12 months (85/673, 9.2% vs 267/1205, 19.6% less than 5 visits; aPR 0.44, 95% CI 0.32-0.59). No differences were found by education, annual household income, health insurance, marital status, location, or census region in the adjusted model. Individuals who reported that the primary reason for their most recent telehealth visit in the past year was for a minor illness or acute care were more likely to be married or cohabitating (322/1053, 36.4% vs 188/825, 25.4% divorced, widowed, separated, or single; aPR 1.30, 95% CI 1-1.68), and less likely to be non-Hispanic Black adults (51/271, 20.9% vs 321/1145, 33.2% White; aPR 0.62, 95% CI 0.41-0.95) and living in a rural location (50/193, 22.1% vs 460/1685, 33.37% urban; aPR 0.64, 95% CI 0.42-0.97). No differences were found by age, sex, education, annual household income, health insurance, census region, or frequency of visits with a health professional in the previous 12 months in the adjusted model.

Individuals who reported that the primary reason for their most recent telehealth visit was for chronic disease management were more likely to be aged between 45 and 64 years and 65 years and older (192/696, 26.1% and 184/560, 28.4% vs 86/622, 13.3% aged between 18 and 44 years; aPR 1.93, 95% CI 1.27-2.93 and aPR 2.08, 95% CI 1.33-3.23) and to have had 5 or more visits with a health professional in the previous 12 months (229/673, 29.9% vs 233/1205, 16.72% less than 5 visits; aPR 1.67, 95% CI 1.23-2.26), and less likely to have an annual household income of ≥US $75,000 (188/875, 17.6% vs 141/466, 29.5% ≤US $34,999; aPR 0.60, 95% CI 0.37-0.98). No differences were found by sex, race or ethnicity, education, health insurance, marital status, location, or census region in the adjusted model. Individuals who reported that the primary reason for their most recent telehealth visit was for mental health, behavioral, or substance abuse issues were less likely to be aged between 45 and 64 years and 65 years and older (110/696, 13.2% and 29/560, 4.7% vs 149/622, 25.5% aged between 18 and 44 years; aPR 0.61, 95% CI 0.45-0.82 and aPR 0.19, 95% CI 0.10-0.35), non-Hispanic Black and Hispanic (24/271, 7.9% and 47/367, 13.5% vs 208/1145, 19.09% White; aPR 0.42, 95% CI 0.24-0.73 and aPR 0.61, 95% CI 0.37-0.99), and married or cohabitating (131/1053, 11.33% vs 157/825, 25.9% divorced, widowed, separated, or single; aPR 0.44, 95% CI 0.28-0.67). No differences were found by sex, education, annual household income, health insurance, location, census region, or frequency of visits with a health professional in the previous 12 months in the adjusted model. Multimedia Appendix 1 shows full details on the predictors of primary reasons for telehealth visits in the 12 months before HINTS 6.

## Discussion

### Summary of Findings

In this nationally representative sample, 38.78% (1986/4830) of US adults had a telehealth visit in the 12 months before March 2022-November 2022. Similarly, 2021 NHIS data demonstrated that 37% of adults had a telehealth visit in the 12 months before January 2021-December 2021 [15]. Thus, telehealth use has remained steady over the past year following telemedicine expansion. While 2021 NHIS data demonstrated significant disparities in telehealth visits by age, race or ethnicity, income, and location [15], this analysis did not.

The 2021 NHIS data demonstrated that young adults were less likely to have a telemedicine visit in the past year [15]. While telehealth visit attendance was similar by age in this analysis, older adults were more likely to report that the primary reason for their most recent telehealth visit was for an annual visit or for chronic disease management. Meanwhile, young adults were more likely to report that their primary reason was mental health, behavioral, or substance abuse issue. The high prevalence of telehealth use for mental health, behavioral, and substance abuse issues among young adults during COVID-19 may explain the narrowing of telehealth disparities by age since 2021. Claims data from January 2020 to June 2020 demonstrated that 57% of psychiatry visits were conducted through telemedicine [26].

The 2021 NHIS data demonstrated that individuals with a lower annual income, living in more rural areas, and non-Hispanic Black, non-Hispanic Asian, and Hispanic adults were less likely to have a telehealth visit in the past year [15]. In this analysis, there were no significant differences in telehealth visit attendance by income, location, or race or ethnicity. COVID-19 restrictions may have forced people of all socioeconomic statuses to move toward telehealth. Additionally, while telehealth was originally intended for individuals living in rural areas, it may also be an important modality for increasing access to care among individuals living in urban settings. While disparities in telehealth use may be narrowing, the impact of telehealth use on patient outcomes remains unclear [27]. Other studies have evaluated national telehealth use [28,29]; however, we primarily compared our findings to 2021 NHIS data due to its similar methodology (ie, it is a nationally representative survey asking about telehealth use in the previous 12 months).

Our findings largely align with those of other studies. For example, the US Census Bureau’s Household Pulse Survey data, from April 14, 2021, through August 8, 2022, found that 23% of adult respondents (N=1,180,248) reported having a telehealth visit within the last 4 weeks [29]. In this study, we observed a higher prevalence of participants having a telehealth visit. It is important to note that HINTS 6 evaluated telehealth use over 12 months, whereas the Household Pulse Survey assessed it over a shorter period of 4 weeks [28,29]. The Pulse Survey found that those who self-reported as Hispanic or Latino, Black, and multiracial or other; aged less than 65 years old; making less than US $100,000 per year; having a bachelor’s degree or higher; with Medicare and Medicaid; and with other insurance types were more likely to have had a telehealth visit [29]. However, individuals who were uninsured and from the Northeast, Midwest, and South regions of the United States were less likely to have had a telehealth visit [29]. In our analysis, we did not find any disparities in telehealth use by age, race or ethnicity, or income. The Pulse Survey actually found that telehealth use may be higher in individuals that have a lower annual household income or are from underrepresented racial and ethnic groups [29]. However, unlike our findings, the Pulse Survey demonstrated that individuals aged 65 years and older were less likely to have reported having a telehealth visit over the previous 4 weeks [29].

Similar to our findings, the Pulse Survey also found that individuals living in the Midwest were less likely to have had a telehealth visit, and individuals with health insurance were more likely to have had a telehealth visit than adults who were uninsured [29]. The Pulse Survey found that approximately 9% of uninsured adults had a telehealth visit in the previous 4 weeks [29] while in HINTS 6, 22% of uninsured adults had a telehealth visit in the past 12 months. This difference may be a reflection of the length of the assessment periods between the 2 studies. Almost a quarter of the uninsured participants in this analysis had a telehealth visit in the past 12 months. While lower than insured individuals, the number of uninsured adults with telehealth visits was still fairly high. Telehealth may present a cheaper option for uninsured individuals than in-person care, saving them money on travel and loss of wages.

The Pulse Survey also evaluated predictors of video versus audio-only telehealth visits from July 21, 2021, to August 8, 2022 [28,29]. Video telehealth usage was lower among Hispanic or Latino, Black, and Asian adults; men; older adults (aged ≥65 years); and adults with less than a bachelor’s degree and an annual household income of less than US $100,000 [29]. While we did not evaluate predictors of video versus audio-only telehealth visits in this analysis, we will explore them in future work. There may be some instances when audio-only telehealth visits, depending on the reason for the telehealth visit, are not sufficient and result in lower quality care than an in-person visit or a video telehealth visit. Patient outcomes should be compared by health problem across in-person, video telehealth, and audio-only telehealth visits in the future.

Having a telehealth visit in the past 12 months for a minor illness or acute care (510/1878, 32.15%) was the most common primary reason for participants’ most recent telehealth visit, followed by managing a chronic disease (462/1878, 21%), mental health, behavioral, or substance abuse issues (288/1878, 16.94%), and an annual visit (352/1878, 16.22%). A review of large-scale claims studies published during the first year of the pandemic found that telehealth use varied widely across specialties and diagnoses, but the highest levels were seen for behavioral health [30]. Meanwhile, a 2020 analysis of Doximity data demonstrated that the top 10 specialties using telemedicine largely treated chronic illnesses [31]. In addition to supporting chronic disease management and mental or behavioral health, telemedicine may play an important role in addressing minor or acute illnesses (eg, fever or sinus infection) in the future. In this study, we also found that individuals, especially those who cited that the primary reason for their most recent telehealth visit was for chronic disease management, with a higher frequency of visits with a health professional, may be more likely to engage in telehealth.

In this analysis, middle-aged and older adults were less likely to report that the primary reason for their most recent telehealth visit was for a mental health, behavioral, or substance abuse issue (110/696, 13.2% aged between 44 and 64 years and 29/560, 4.7% aged ≥65 years, vs 149/622, 25.5% aged between 18 and 44 years). However, according to 2019 NHIS data, the prevalence of experiencing any level of depressive symptoms is similar across age groups (approximately 18%) [32]. While mental health issues are prevalent in older adults, this population may be more likely to schedule telehealth visits for chronic disease management. In this analysis, non-Hispanic Black and Hispanic adults were also less likely than non-Hispanic White adults to report that the primary reason for their most recent telehealth visit was due to a mental health, behavioral, or substance abuse issue. However, 2019 NHIS data also demonstrated that the prevalence of experiencing any level of depressive or anxiety symptoms was similar across these 3 racial or ethnic groups [32]. Additional research is needed to examine the reason for these disparities in telehealth use for mental health, behavioral, and substance abuse issues.

### Limitations and Strengths

Limitations include the inability to determine causation; the potential for selection bias due to the HINTS 6 low response rate (28% [7], consistent with declining rates across many national surveys [33]) and recall bias, the combining of audio-only and audio-video telehealth visits, and not having data on health insurance plan type. However, the survey weighting was calibrated to adjust for demographic variables by response rate. We will explore predictors of audio-only and audio-video telehealth visits separately in future work. Despite these limitations, HINTS 6 is the first national study to ask about telehealth use over 12 months during the latter stages of COVID-19. Furthermore, this study also looked beyond the prevalence of telehealth visits to the prevalence and predictors of participants’ primary reasons for their most recent telehealth visit.

### Conclusions

Telehealth visit attendance, over a year following telemedicine expansion in response to COVID-19, remained high (1986/4830, 38.78%). Disparities in telehealth visit attendance by age, race or ethnicity, income, and location may be less pronounced than at the beginning of COVID-19. There may be continued opportunities to leverage telehealth following the pandemic to increase access to care and promote health equity.

## Appendix

Multimedia Appendix 1 Predictors of primary reasons for telehealth visits in 12 months before Health Information National Trends Survey 6 (March 2022-November 2022) among weighted US adults (n=1878).

## Abbreviations

ACS: American Community Survey
aPR: adjusted prevalence ratio
HINTS 6: Health Information National Trends Survey
NHIS: National Health Interview Survey

## Data Availability

The HINTS 6 data set analyzed in this study are made publicly available by the National Cancer Institute [34].
