# Supplementary material for: Prevalence and Disparities in Telehealth Use Among US Adults Following the COVID-19 Pandemic: National Cross-Sectional Survey
Source: J Med Internet Res. 2024 May 10;26:e52124. doi: 10.2196/52124 (PMC11127137; doi:10.2196/52124)
Supplement: Multimedia Appendix 1 [file jmir_v26i1e52124_app1.pdf]

Table S1. Predictors of primary reasons for telehealth visits in 12 months before Health Information National Trends Survey 6 (March 2022-November 2022) among weighted US adults (n=1878).

	**Reasons for Telehealth Visit, Unadjusted/Adjusted Prevalence Ratios (95% CI)**							
	**Annual Visit**		**Minor Illness/Acute Care**		**Chronic Disease Management**		**Mental Health/Substance Abuse**	
**Sociodemographic Characteristics**	**Unadjusted PR (95% CI)**	**Adjusted PR (95% CI)**	**Unadjusted PR (95% CI)**	**Adjusted PR (95% CI)**	**Unadjusted PR (95% CI)**	**Adjusted PR (95% CI)**	**Unadjusted PR (95% CI)**	**Adjusted PR (95% CI)**
Age, years								
18-44	1 (Ref)	1 (Ref)	1 (Ref)	1 (Ref)	1 (Ref)	1 (Ref)	1 (Ref)	1 (Ref)
45-64	0.88 (0.58-1.32)	0.86 (0.57-1.29)	1.10 (0.84-1.43)	1.05 (0.78-1.40)	**1.96 (1.32-2.90)***	**1.93 (1.27-2.93)***	**0.52 (0.37-0.73)****	**0.61 (0.45-0.82)***
65+	**1.96 (1.38-2.77)****	**2.16 (1.52-3.08)****	**0.71 (0.56-0.97)***	0.72 (0.51-1.02)	**2.13 (1.42-3.21)****	**2.08 (1.33-3.23)***	**0.18 (0.10-0.35)****	**0.19 (0.10-0.35)****
Sex								
Male	1 (Ref)	1 (Ref)	1 (Ref)	1 (Ref)	1 (Ref)	1 (Ref)	1 (Ref)	1 (Ref)
Female	**0.69 (0.50-0.95)***	**0.68 (0.50-0.93)***	0.94 (0.75-1.19)	1.00 (0.78-1.27)	1.01 (0.76-1.33)	0.97 (0.73-1.28)	1.45 (0.82-2.58)	1.49 (0.91-2.46)
Race/Ethnicity								
NH White	1 (Ref)	1 (Ref)	1 (Ref)	1 (Ref)	1 (Ref)	1 (Ref)	1 (Ref)	1 (Ref)
NH Black	**1.83 (1.24-2.71)***	**1.96 (1.28-3.00)***	**0.63 (0.41-0.96)***	**0.62 (0.41-0.95)***	1.44 (0.88-2.35)	1.34 (0.85-2.10)	**0.41 (0.24-0.70)***	**0.42 (0.24-0.73)***
Hispanic	0.77 (0.52-1.12)	0.78 (0.49-1.22)	0.97 (0.74-1.28)	0.94 (0.68-1.29)	1.19 (0.80-1.78)	1.39 (0.85-2.26)	0.71 (0.44-1.14)	**0.61 (0.37-0.99)***
NH Asian	1.22 (0.68-2.17)	1.25 (0.70-1.73)	1.14 (0.77-1.68)	1.04 (0.71-1.51)	1.17 (0.57-2.41)	1.60 (0.80-3.21)	0.88 (0.33-2.37)	0.63 (0.30-1.36)
Education								
High School Graduate or Less	1 (Ref)	1 (Ref)	1 (Ref)	1 (Ref)	1 (Ref)	1 (Ref)	1 (Ref)	1 (Ref)
Some College/ Vocational or Technical School	0.95 (0.57-1.60)	0.96 (0.60-1.53)	1.04 (0.67-1.61)	0.94 (0.60-1.45)	1.25 (0.81-1.93)	1.39 (0.92-2.09)	0.71 (0.38-1.31)	0.77 (0.46-1.31)
College Graduate/ Postgraduate	1.10 (0.77-1.57)	1.15 (0.76-1.73)	1.07 (0.71-1.59)	0.87 (0.57-1.35)	0.99 (0.66-1.46)	1.33 (0.90-1.98)	0.85 (0.52-1.39)	0.73 (0.43-1.23)
Income								
≤$34,999	1 (Ref)	1 (Ref)	1 (Ref)	1 (Ref)	1 (Ref)	1 (Ref)	1 (Ref)	1 (Ref)
$35,000-$74,999	0.84 (0.52-1.38)	0.71 (0.43-1.17)	1.44 (0.85-2.42)	1.34 (0.76-2.37)	0.71 (0.45-1.12)	0.70 (0.45-1.10)	1.17 (0.63-2.19)	1.51 (0.78-2.95)
≥$75,000	0.86 (0.57-1.30)	0.71 (0.42-1.20)	**1.68 (1.03-2.75)***	1.38 (0.81-2.35)	**0.60 (0.40-0.88)***	**0.60 (0.37-0.98)***	0.99 (0.64-1.53)	1.67 (0.96-2.92)
Health Insurance								
No	1 (Ref)	1 (Ref)	1 (Ref)	1 (Ref)	1 (Ref)	1 (Ref)	1 (Ref)	1 (Ref)
Yes	1.18 (0.60-2.35)	0.97 (0.49-1.90)	1.16 (0.73-1.82)	1.23 (0.76-2.01)	1.10 (0.55-2.17)	0.87 (0.44-1.74)	0.81 (0.24-2.68)	1.01 (0.34-3.03)
Marital Status								
Divorced/Widowed/ Separated/Single	1 (Ref)	1 (Ref)	1 (Ref)	1 (Ref)	1 (Ref)	1 (Ref)	1 (Ref)	1 (Ref)
Married/ Cohabitating	1.13 (0.75-1.70)	1.27 (0.77-2.08)	**1.43 (1.07-1.91)***	**1.30 (1.00-1.68)***	1.01 (0.79-1.29)	1.04 (0.85-1.26)	**0.44 (0.30-0.65)****	**0.44 (0.28-0.67)****
Location								
Urban	1 (Ref)	1 (Ref)	1 (Ref)	1 (Ref)	1 (Ref)	1 (Ref)	1 (Ref)	1 (Ref)
Rural	0.87 (0.53-1.41)	0.89 (0.54-1.47)	**0.66 (0.45-0.96)***	**0.64 (0.42-0.97)***	**1.53 (1.05-2.22)***	1.39 (0.93-2.09)	1.16 (0.69-1.94)	1.35 (0.80-2.78)

Census Region								
Northeast	1 (Ref)	1 (Ref)	1 (Ref)	1 (Ref)	1 (Ref)	1 (Ref)	1 (Ref)	1 (Ref)
Midwest	0.96 (0.54-1.71)	1.03 (0.56-1.88)	1.15 (0.78-1.70)	1.23 (0.85-1.78)	1.36 (0.81-2.26)	1.16 (0.71-1.88)	0.69 (0.38-1.26)	0.65 (0.37-1.17)
South	0.86 (0.55-1.33)	0.77 (0.49-1.21)	1.09 (0.80-1.49)	1.20 (0.85-1.68)	1.39 (0.88-2.17)	1.25 (0.82-1.90)	0.67 (0.35-1.28)	0.75 (0.39-1.45)
West	0.97 (0.57-1.64)	0.95 (0.54-1.66)	1.16 (0.83-1.61)	1.16 (0.82-1.64)	1.28 (0.77-2.13)	1.28 (0.79-2.07)	0.82 (0.47-1.44)	0.88 (0.52-1.51)
Frequency of Visits with Health Professional								
<5	1 (Ref)	1 (Ref)	1 (Ref)	1 (Ref)	1 (Ref)	1 (Ref)	1 (Ref)	1 (Ref)
≥5	**0.47 (0.35-0.63)****	**0.44 (0.32-0.59)****	**0.75 (0.59-0.96)***	0.78 (0.60-1.01)	**1.79 (1.34-2.38)****	**1.67 (1.23-2.26)***	1.24 (0.85-1.83)	1.35 (0.93-1.97)

Weights are calibrated using data from the 2021 American Community Survey (age, sex, educational attainment, marital status, race, ethnicity, and census region) conducted by the U.S. Census Bureau

Adjusted PR Model: adjusting for age, sex, race/ethnicity, education, income, health insurance, marital status, location, census region, and frequency of visits with a health professional

* P<0.05; ** P<0.001
